# Non-wetting surface-driven high-aspect-ratio crystalline grain growth for efficient hybrid perovskite solar cells

**DOI:** 10.1038/ncomms8747

**Published:** 2015-07-20

**Authors:** Cheng Bi, Qi Wang, Yuchuan Shao, Yongbo Yuan, Zhengguo Xiao, Jinsong Huang

**Affiliations:** 1 Department of Mechanical and Materials Engineering, College of Engineering, University of Nebraska-Lincoln, Lincoln, Nebraska 68588-0656, USA; 2 Nebraska Center for Materials and Nanoscience, University of Nebraska-Lincoln, Lincoln, Nebraska 68588-0656, USA

## Abstract

Large-aspect-ratio grains are needed in polycrystalline thin-film solar cells for reduced charge recombination at grain boundaries; however, the grain size in organolead trihalide perovskite (OTP) films is generally limited by the film thickness. Here we report the growth of OTP grains with high average aspect ratio of 2.3–7.9 on a wide range of non-wetting hole transport layers (HTLs), which increase nucleus spacing by suppressing heterogeneous nucleation and facilitate grain boundary migration in grain growth by imposing less drag force. The reduced grain boundary area and improved crystallinity dramatically reduce the charge recombination in OTP thin films to the level in OTP single crystals. Combining the high work function of several HTLs, a high stabilized device efficiency of 18.3% in low-temperature-processed planar-heterojunction OTP devices under 1 sun illumination is achieved. This simple method in enhancing OTP morphology paves the way for its application in other optoelectronic devices for enhanced performance.

## Abstract (web summary)

The performance of hybrid perovskite solar cells is diminished by charge recombination, which commonly occurs at grain boundaries. Here, the authors employ a non-wetting hole transport layer which promotes the growth of highly crystalline films with fewer grain boundaries, leading to improved device efficiencies.

## Graphical abstract

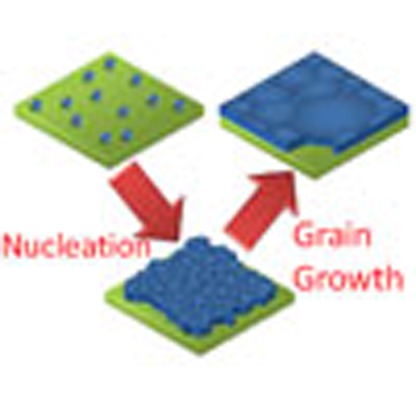

Organolead triiodide perovskite (OTP)-based solar cells have been attracting increasing attention from the solar energy community in the past few years due to the rapid increase of device power conversion efficiency (PCE) to the level of silicon solar cells and low-cost prospectus of this technology in terms of raw materials and device fabrication^1^,^2^,^3^,^4^,^5^,^6^,^7^,^8^,^9^,^10^,^11^. Nearly every important progress in device efficiency enhancement counts on the improved material quality of the OTP films for both mesoporous structure and planar heterojunction (PHJ) structure OTP devices^1^,^2^,^3^,^12^,^13^,^14^,^15^,^16^,^17^. Similar to any polycrystalline thin film solar cells, larger OTP grains with less grain boundaries have been shown to increase the efficiency of OTP devices^3^,^12^. More evidences have shown that grain boundaries in OTP films might cause increasing charge recombination due to the presence of large density of charge traps^4^,^13^,^14^,^18^,^19^,^20^. Our recent studies have shown that the carrier diffusion length can be boosted to record value of above 200 μm under 1 sun illumination and larger than 3 mm under weak light at room temperature for both electrons and holes in OTP single crystals that have no grain boundaries^14^. Although it is plausible to develop record high efficiency device with the OTP single crystals, the increasing material cost and lacking of techniques to scale up OTP single-crystal growth disgrace its prospectus for large-area, low-cost solar panels. An ideal OTP solar cell should have thin OTP layers with thickness sufficient to absorb most of the sun light, which is around 300–600 nm thanks to the very high extinction efficiency of OTP materials^6^, and with grain size as large as the cells to simulate the single-crystal solar cells. However, the size of the OTP grains grown by many methods is generally limited by the film thickness of the OTP films^3^,^12^,^21^.

A trick to grow large-size clear single-crystal ices is to use clean, smooth and non-wetting plastic container to prevent the formation of too dense nuclei from heterogeneous nucleation. In this manuscript, we used the same trick to grow OTP films with large crystalline grains on a wide range of non-wetting hole transport layers (HTLs). The average grain size/thickness aspect ratio in the OTP films grown on non-wetting HTLs reaches 2.3–7.9, which dramatically reduces charge trap density by 10–100-fold and boosts the PCE of OTP planar heterojunction solar cells to 18.3%.

## Results

### Mechanism of OTP grain growth on non-wetting surfaces

**Figure 1:**
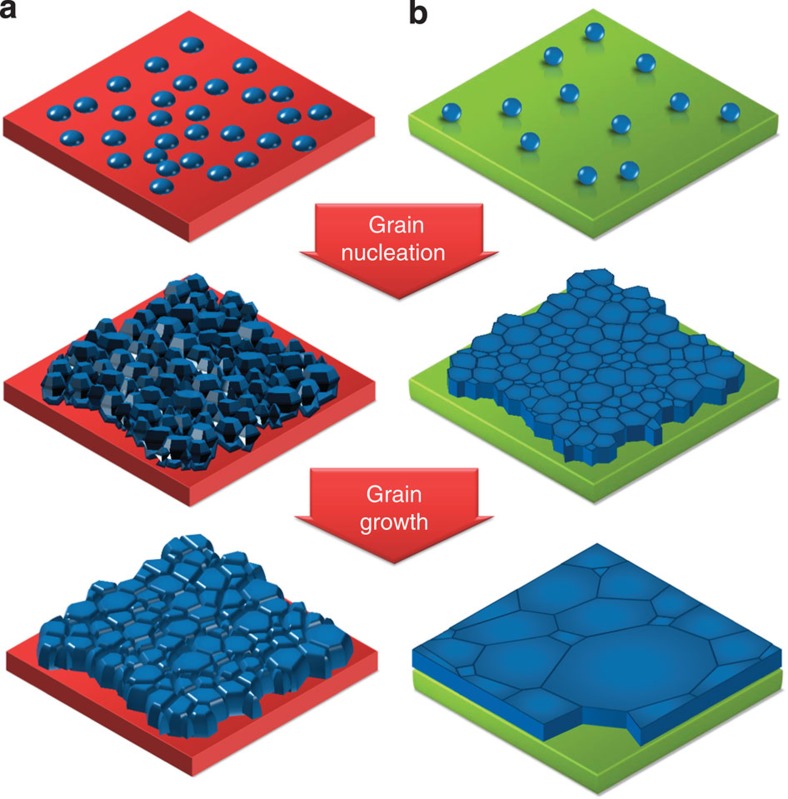
Mechanism of large grain growth on non-wetting HTLs. Illustration of the nucleation and growth of the grains on wetting (**a**) and non-wetting HTLs (**b**) after thermal annealing.

The mechanism of growing large-size OTP grain on non-wetting surfaces is illustrated in Fig. 1, which shows the difference of nucleation and grain growth process on a wetting and a non-wetting surface. Here the two-step thermal annealing-assisted interdiffusion method was employed that forms continuous and pinhole-free OTP films^12^. The first step of OTP film formation is OTP nucleation on the substrates after the chemical reaction of PbI_2_ and MAI (MA is methylammonium). Then the followed drying and thermal annealing processes drive the interdiffusion and complete reaction of the two precursors, which forms and grows the OTP grains. It has been shown that thermal annealing increases the OTP grain size to above 300 nm on the wetting surface of poly(3,4-ethylenedioxythiophene) polystyrene sulfonate (PEDOT:PSS), after 2 h annealing at 105 °C, while further increasing of thermal annealing time causes the decomposition of OTP films without significantly increasing the grain size. It indicates the grain boundaries were pinned, most likely, by the impurities lying in the grain boundaries^22^. Adding solvent vapour during thermal annealing can de-pin the grain boundary and drive larger grain formation, however, the average grain size in OTP films grown on PEDOT:PSS is still limited to be the film thickness^12^. Another feature of the solvent-annealed OTP films is the presence of some small grains close to PEDOT:PSS side and tilted grain boundaries between the large grains, as shown in cross-section scanning electron microscopy (SEM) image in Supplementary Fig. 1. This can be explained by the surface tension dragging force from the wetting PEDOT:PSS substrates, which reduces the grain boundary mobility. Such dragging force dramatically diminishes if the substrate is non-wetting and smooth, yielding a higher grain boundary mobility, which enables the growth of larger grains. The very smooth surface of the polymer HTL also suppresses the nucleation in small cavity and contributes to the high grain boundary mobility.

### Large aspect ratio OTP grain growth on non-wetting HTLs

**Figure 2:**
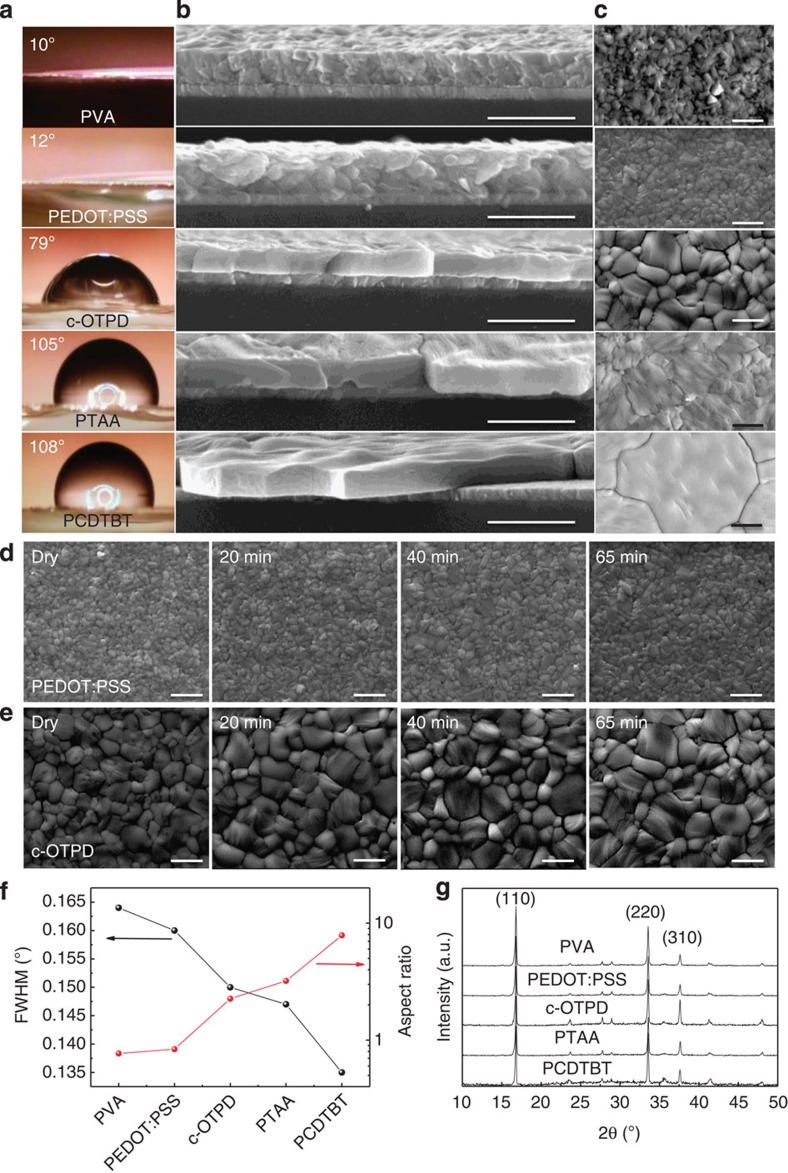
Morphology of MAPbI_3_ films grown on wetting and non-wetting HTLs. The contact angle of water on the varied HTLs (**a**), the cross-section SEM (**b**), top-view SEM (**c**) and X-ray diffraction patterns of the 360-nm MAPbI_3_ grown on PVA-, PEDOT:PSS-, c-OTPD-, PTAA- and PCDTBT-covered ITO substrates (**g**). Scale bars, 1 μm in **b**,**c**; (**d**,**e**) the top-view SEM images of the MAPbI_3_ grown on PEDOT:PSS (top row) and c-OTPD (bottom row) right after drying and after 20, 40 and 65 min of thermal annealing at 105 °C. Scale bar, 1 μm; (**f**) HTL-dependent X-ray diffraction (110) peak full width at half maximum (FWHM) and average grain size/thickness aspect ratio of the MAPbI_3_.

The proposed grain growth mechanism was demonstrated by studying the grain morphology of methylammonium lead triiodide (MAPbI_3_) films on a wide range of wetting and non-wetting polymer substrates, including polyvinyl alcohol (PVA), PEDOT:PSS, crosslinked *N*4,*N*4′-bis(4-(6-((3-ethyloxetan-3-yl)methoxy)hexyl)phenyl)-*N*4,*N*4′-diphenylbiphenyl-4,4′-diamine (c-OTPD), poly(bis(4-phenyl)(2,4,6-trimethylphenyl)amine) (PTAA) and poly(*N*-9′-heptadecanyl-2,7-carbazole-alt-5,5-(4′,7′-di-2-thienyl-2′,1′,3′-benzothiadiazole)) (PCDTBT). The chemical structures of these polymers are shown in Supplementary Fig. 2. The hydroxyl group in PVA and PEDOT:PSS provides wetting surfaces, while other polymers are hydrophobic. The presence of oxygen group in c-OTPD makes it less hydrophobic as PTAA or PCDTBT. The contacting angles of water on these polymers shown in Fig. 2a are 10°, 12°, 79°, 105° and 108° for PVA, PEDOT:PSS, c-OTPD, PTAA and PCDTBT, respectively. The measured contact angle measured here represents the wetting capability of the different substrate surfaces to water, and we hypothesize it is also an indication of the wetting capability of these surfaces to solid-state OTP because of its hydrophilic property, as it can be dissolved in many polar solvents like *N*,*N*-dimethylformamide (DMF). Solution process was used to fabricate the HTLs and the MAPbI_3_ layer, where the HTL was first spun, and then some were crosslinked by ultraviolet curing and thermal annealing. The fabrication details of the HTLs are shown in the Methods section. The MAPbI_3_ film was fabricated by thermal annealing-induced interdiffusion method^5^,^10^,^12^. The hydrophobic surfaces of non-wetting HTLs imposed a challenge in fabricating continuous pinhole-free hydrophilic OTP films on them although it is good for the large grain growth. Formation of continuous PbI_2_ films on the HTLs is a prerequisite for the continuous OTP film formation. To form continuous PbI_2_ on the HTLs, the PbI_2_ solution was heated to 110 °C and quickly dripped on the HTLs for spin coating, which yielded smooth PbI_2_ films over the whole substrate as shown by the optical microscope images in Supplementary Fig. 3a. At lower solution temperature, PbI_2_ solution formed non-uniform films with many spots, as shown in Supplementary Fig. 3b. Here all the MAPbI_3_ films were thermal annealed for 65 min at 105 °C. The cross-section and top-view SEM images of the MAPbI_3_ films on these HTLs shown in Fig. 2b,c reveal a clear correlation between the substrate surface wetting capability and the grain morphology: First, the average grain size is much larger for MAPbI_3_ films on hydrophobic HTLs. The grain size distribution for MAPbI_3_ on various HTLs was summarized in Supplementary Fig. 4. The sizes of MAPbI_3_ grains on wetting PVA (277 nm) and PEDOT:PSS (301 nm) are smaller than the film thickness (∼360 nm) after 65 min of thermal annealing. Most grains in MAPbI_3_ films on non-wetting HTLs were much larger than the film thickness, with the average grain size/film thickness aspect ratio reaching 2.3, 3.2 and 7.9 for c-OTPD, PTAA and PCDTBT (Fig. 2f), respectively. The largest grain size on PCDTBT reached ∼5 μm, which is about 14-fold of the film thickness; second, most of the grain boundaries are perpendicular to the substrate for films on non-wetting HTLs to minimize the grain boundary energy. It is noted that PCDTBT is so hydrophobic so that the OTP films on PCDTBT were mostly not continuous, and SEM pictures were taken from areas with OTP films.

The influence of surface tension force on the grain nucleation and growth was verified by comparing grain morphology evolution of the MAPbI_3_ films on two HTLs, wetting PEDOT:PSS and non-wetting c-OTPD. As shown by the SEM images in Fig. 2d,e, the average size of the MAPbI_3_ grains on c-OTPD (480 nm) is much larger than that on PEDOT:PSS (230 nm) just after film drying, verifying that a non-wetting surface suppresses heterogeneous nucleation, results in less dense nuclei and thus larger grain size. The average size of the MAPbI_3_ grains on c-OTPD increased to >800 nm with the largest one reaching >1.5 μm after 65 min of thermal annealing. In contrast, the average grain size for the MAPbI_3_ film on PEDOT:PSS remained to be around 300 nm even after 65 min of thermal annealing. Finally, the HTLs also impact the crystallinity of the OPT films formed on them. The X-ray diffraction peaks became stronger and sharper for perovskite films on hydrophobic HTLs, agreeing with disappearance of the small grains and better film crystallinity (Fig. 2f,g). No significant change of the diffraction peak ratio was observed, indicating the different HTLs do not cause crystal orientation change.

### Influence of the grain aspect ratio on device performance

**Figure 3:**
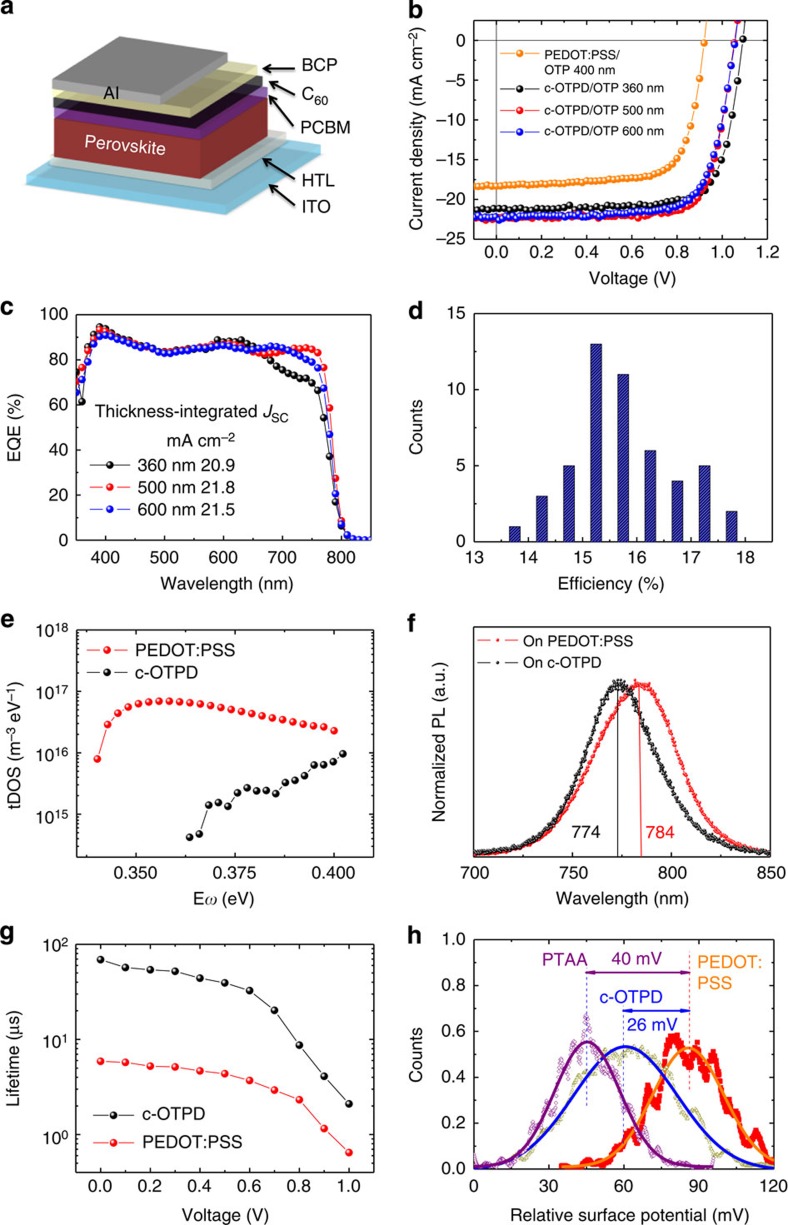
Characterization of the MAPbI_3_ devices with PEDOT:PSS and c-OTPD HTLs. (**a**) The PHJ device structure; (**b**) *J*–*V* of the devices with PEDOT:PSS and c-OTPD HTLs, and with different MAPbI_3_ thickness of 360, 500 and 600 nm; (**c**) EQE of the devices with c-OTPD HTL and with different MAPbI_3_ thickness of 360, 500 and 600 nm; (**d**) efficiency histogram of the 50 devices using c-OTPD as HTL; (**e**) tdOS, (**f**) photoluminescence at room temperature and (**g**) impedance spectroscopy lifetime at different bias for the devices with PEDOT:PSS and c-OTPD HTLs. (**h**) Work function distribution of the HTLs of PEDOT:PSS, c-OTPD and PTAA.

The influence of the increased grain size and crystallinity on the device performance was evaluated in PHJ devices with a structure shown in Fig. 3a. Again, c-OPTD and PEDOT:PSS devices were studied because of the excellent reproducibility of the device performance using these two HTLs. Figure 3b shows the photocurrent–voltage (*J*–*V*) curves of the optimized MAPbI_3_ devices using c-OTPD and PEDOT:PSS as HTLs. The device with PEDOT:PSS as HTL has a relatively low efficiency of 12.3%, which is consistent with previous result for the devices using only thermal annealing^22^. The device's short circuit current density (*J*_SC_) increased from 18.6 to 21.2 mA cm^−2^ and open circuit voltage (*V*_OC_) increased significantly from 0.92 to 1.09 V, when the device's HTL was changed from PEDOT:PSS to c-OTPD. The device employing c-OTPD showed a decent fill factor (FF) of 75.5%, yielding a PCE of 17.5%. Negligible photocurrent hysteresis was observed by changing the scanning directions, as shown in Supplementary Fig. 5. The absence of obvious photocurrent hysteresis is commonly observed in our PHJ devices because the MAPbI_3_ films have large grains, and charge traps at the film surface and grain boundaries are well passivated by fullerenes^10^,^12^,^22^,^23^. The devices with thicker MAPbI_3_ film (500–600 nm) were fabricated to optimize the PCE. A thicker MAPbI_3_ active layer at 500 nm can produce a larger *J*_SC_ of 22.4 mA cm^−2^, resulting in an increased PCE of 17.8%, despite of a decrease of *V*_OC_ to 1.05 V. Further increasing the film thickness did not increase the *J*_SC_ but lowered the FF, which yielded a comparable but slightly lower efficiency of 17.2%, as shown in Fig. 3b. The device performance with varied HTLs, OTPs and OTP film thicknesses is summarized in Table 1. The peak external quantum efficiency (EQE) of the c-OTPD device with 360-nm-thick MAPbI_3_ reached 94.5% at 390 nm, as shown in Fig. 3c, and the calculated *J*_SC_ from EQE for the three devices are in good agreement to measured *J*_SC_. The increased *J*_SC_ (21.8 and 21.5 mA cm^−2^) in the device with 500- and 600-nm-thick MAPbI_3_ should mostly be ascribed to the improved absorption in the wavelength range of 650–800 nm. The efficiency histogram in Fig. 3d shows >35% of the devices the PCE higher than 16.0%, and >80% devices have PCE higher than 15.0%.

## Discussion

To find the correlation between the device PCE enhancement and larger grain size and better crystallinity, we performed thermal admittance spectroscopy (TAS) measurement to examine the trap density-of-states (tDOS) in the devices with two different HTLs, PEDOT:PSS and c-OTPD. Our previous study showed tDOS of the devices with PEDOT:PSS had a tDOS in the order of 10^16^ m^−3^ eV^−1^ in the trap band of 0.310–0.425 eV towards the forbidden gap, which is correlated to grain boundaries^4^,^12^. As shown in Fig. 3e, the device with c-OTPD HTL had a 10–100-fold smaller tDOS of 10^14^–10^15^ m^−3^ eV^−1^. It should be noticed that this trap density is as low as those measured in single crystals using TAS characterization^14^. The much lower tDOS can be partially explained by significant enlarged MAPbI_3_ grain size on c-OTPD (Fig. 2b,c). However, it is noted that MAPbI_3_ grain size on c-OTPD is 2.7-fold that on PEDOT:PSS, which yields only 2.7 times less grain boundary area. If the specific tDOS in unit grain boundary area is the same for the films on both HTLs, there should be additional factors contributed the dramatically reduced tDOS. We speculate that the improved quality of c-OTPD/MAPbI_3_ interface also contributes to the tDOS reduction. This speculation is supported by the observed large peak EQE of 94.5% at the 390 nm in the device with c-OTPD, which is much higher than the devices with PEDOT:PSS^10^,^12^,^13^. Since shorter-wavelength light has shorter penetration depth in OTPs, the generated charges are more susceptible to charge recombination at the HTL/MAPbI_3_ interface. A high EQE above 90% at shorter wavelength proves the very good passivation of charge traps in MAPbI_3_ bottom surface close to the transparent electrode side.

The reduction of tDOS at MAPbI_3_ bottom surface (c-OTPD side) was further confirmed by photoluminescence study. In our previous study of passivation of MAPbI_3_ top surface by fullerenes, a blue shift of photoluminescence was observed for the fullerene-passivated MAPbI_3_ when exciting light was from the top side, while there was no photoluminescence blue shift if the excitation light was from the bottom side, because fullerenes cannot reach the bottom surface of MAPbI_3_ even after 1 h thermal annealing. Here we observed a blue shift of photoluminescence peak from 784 to 774 nm for the c-OTPD/MAPbI_3_ films with incident light from indium tin oxide (ITO) side, as shown in Fig. 3f, confirming the scenario^4^,^12^. The absence of charge traps at MAPbI_3_ bottom surface can be explained by (1) the absence of small MAPbI_3_ grains close to c-OTPD and (2) excellent crystallinity and stoichiometry of the bottom surface. The large surface energy-driven interaction pushes any residual ions to merge to large grains, resembling the hydrophobic interaction that was used for single-crystal nanoparticle synthesis^24^. We speculate the vertical and straight grain boundaries facilitate the diffusion of fullerenes, allowing them move towards the bottom surface, which reduced the trap density near the bottom side.

Now that both top and bottom surfaces of the MAPbI_3_ thin films have low trap density, which resembles the passivation of silicon wafer at all surfaces by oxidization, the charge recombination lifetime (*τ*_r_) should be significantly elongated. As shown in Fig. 3g, the *τ*_r_ measured by impedance spectroscopy is significantly prolonged by 7–10 times in the bias region of 0–0.7 V after replacing PEDOT:PSS with c-OTPD. *τ*_r_ in c-OTPD device at 0 V reached 69 μs, which is close to that of MAPbI_3_ single crystals^14^. The carrier diffusion length in these large crystalline grains should be comparable to that in MAPbI_3_ single crystals, which was demonstrated to exceed 175 μm under 1 sun illumination because of the same carrier recombination lifetime and mobility^4^,^14^, which is already more than 100 times longer than the film's thickness. Therefore, all the photogenerated charges can diffuse to the charge transport layers or grain boundaries without recombination, and the device efficiency is only determined by the charge recombination at grain boundaries or at electrode interfaces. It clearly demonstrated that excellent capability of non-wetting c-OTPD HTL in reducing trap density and suppressing charge recombination in MAPbI_3_. A longer *τ*_r_ is expected to contribute to the observed increase of *J*_SC_, *V*_OC_ and FF, while partial of the increased *V*_OC_ should be ascribed to a higher work function of c-OTPD than PEDOT:PSS. The work function of different HTLs was directly compared by Kelvin probe force microscopy measurement. Gold films deposited on both c-OTPD and PEDOT:PSS surface in the same batch were used as work function reference. As shown in Fig. 3h and Supplementary Fig. 6, a higher work function by 25 meV for c-OTPD than PEDOT:PSS can be derived. The work function difference of the HTLs is much smaller than *V*_OC_ improvement, indicating that the contribution from the reduced charge recombination due to the larger and better OTP grains formed on the non-wetting surface dominates the *V*_OC_ enhancement observed here. This conclusion is further supported by the a recent observation by Kim and coworkers^25^ that a HTL with work function of 5.4 eV, which is higher than ours, was applied in a same device structure with ours, while the highest *V*_OC_ was only 0.98 V. The much lower *V*_OC_ in that work can be explained by the very small grain sizes.

**Figure 4:**
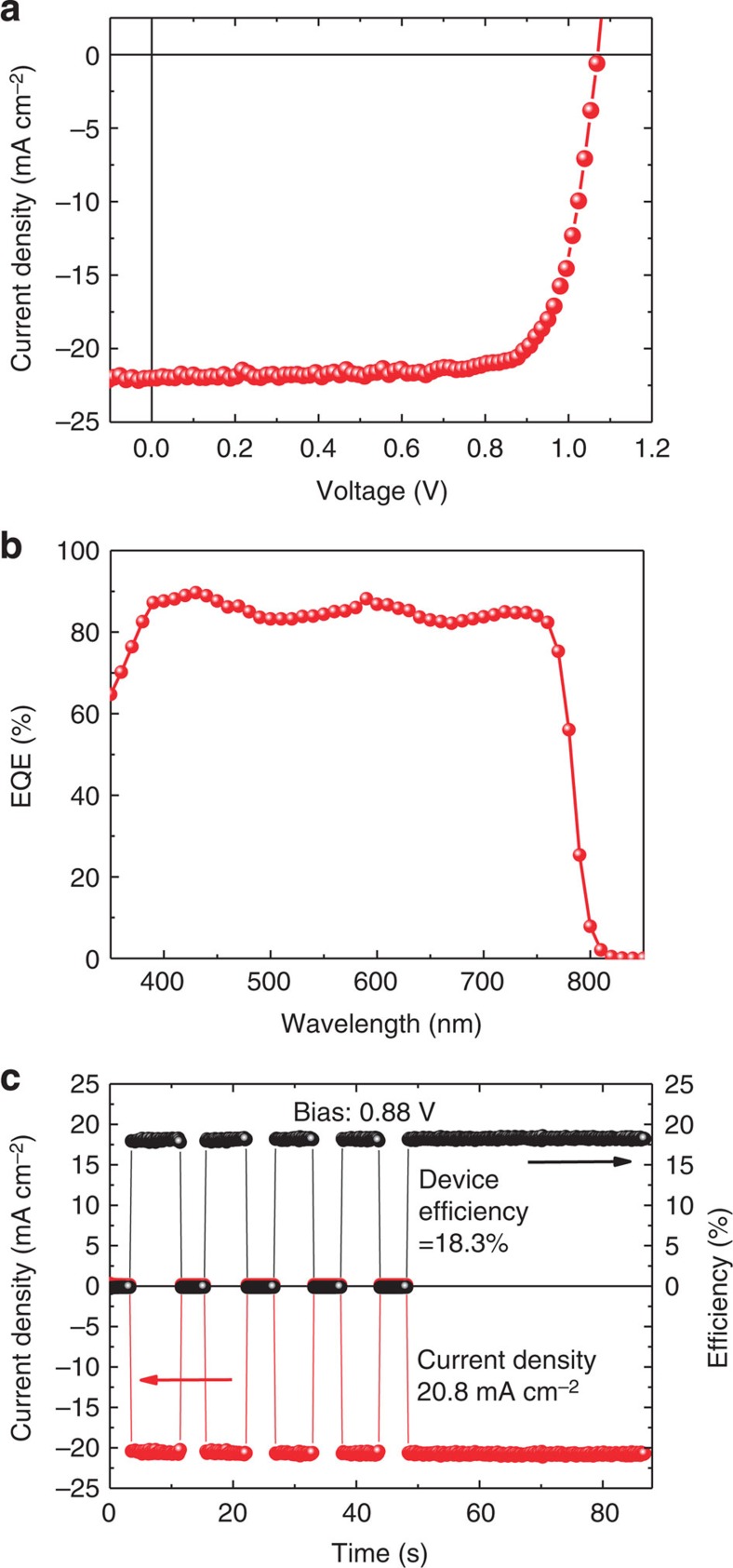
Performance of the MAPbI_3_ devices with PTAA HTL. (**a**) The photocurrent of the MAPbI_3_ devices under 1 sun illumination; (**b**) EQE of the devices with the active layers of MAPbI_3_; (**c**) stabilized photocurrent measurement of the MAPbI_3_ device with PTAA HTL under 1 sun illumination with light turned on and off by a shutter.

It is noted that PTAA has larger OTP grains on it and a slightly higher work function than c-OTPD, therefore we finally optimized our device performance using PTAA HTL. Although larger OTP grains were observed on PCDTBT, most OTP films on PCDTBT have low film coverage <100%, as shown in Supplementary Fig. 7, and thus not suitable for device optimization. The PTAA layer was doped by 1 wt% tetrafluoro-tetracyanoquinodimethane (F4-TCNQ) to enhance its conductivity. Here MAPbI_3_ with a thickness of 500 nm by the two-step interdiffusion method was fabricated. The optimized device performances are shown in Fig. 4a, and are summarized in Table 1. Compared with the device with c-OTPD, the MAPbI_3_ device with PTAA showed a slightly higher efficiency of 18.1% with comparable *V*_OC_ of 1.07 V and *J*_SC_ of 22.0 mA cm^−2^ but a larger FF of 76.8%. The calculated *J*_SC_ from EQE in Fig. 4b reached 21.6 mA cm^−2^, which is in good agreement with measured photocurrent. The photocurrent measured at 0.88 V slightly increased from initial 20.5 to 20.8 mA cm^−2^ (stabilized) after 60-s illumination, resulting in an increased stabilized PCE of 18.3% for the device with PTAA HTL (Fig. 4c). The quick turning on the photocurrent confirms the absence of large density charge traps, while the slow climbing of the *J*_SC_ to the maximum value indicates the presence a relatively small density of deep traps in the devices despite of the passivation of traps on perovskite surface and grain boundaries by the double fullerene layer, which might originate from the deep traps in the fullerene layer itself.

In summary, the non-wetting HTLs were demonstrated to be effective in enhancing the efficiency of OTP PHJ devices to 18.3%. In addition to the higher PCE, these HTLs are expected to be much more stable than the acidic PEDOT:PSS. This work provided a simple method of achieving high-aspect, low-defect density, high-quality perovskite polycrystalline thin films, which possess much better optoelectronic properties such as fewer bulk and surface traps, and higher carrier mobilities. The improved property can potentially give rise to the applications in other fields, such as high mobility for transistors, higher responsivity and lower pink noise for photodetectors by removing the charge traps^26^, lower driving voltage for light emitting diodes and lower threshold excitation density and higher efficiency for lasers.

## Methods

### HTL and OTP layer fabrication

To prepare the OTPD solution for spin coating, diphenyliodonium-hexafluorophosphate was added into OTPD solution with a weight ratio of 2–5 wt% to OTPD as photoinitiator. For c-OTPD film fabrication, 0.25 wt% OTPD solution was first spun on the ITO substrate at 6,000 r.p.m. for 35 s, and then the as-prepared OTPD film was crosslinked by ultraviolet curing (wavelength of 365 nm) for 3 min. To promote the crosslinking, the ultraviolet cured c-OTPD film was thermally annealed at 110 °C for 10 min. PTAA film was prepared by spin coating 0.5 wt% PTAA solution doped with 1 wt% F4-TCNQ at 6,000 r.p.m., and the as-prepared film thermally annealed at 110 °C for 10 min. PCDTBT was prepared by spin coating 0.5 wt% PCDTBT solution at 6,000 r.p.m., and the as-prepared film thermally annealed at 110 °C for 10 min. PEDOT:PSS (Baytron-P 4083) and 1 wt% PVA solution was spin coated on clean ITO substrate at a speed of 3,000 r.p.m. The films were then annealed at 130 °C for 30 min.

The MAPbI_3_ films were fabricated by thermal annealing-induced interdiffusion method^10^. The PbI_2_ layers on all HTLs were spin coated from 110 °C pre-heated PbI_2_ solution in DMF. PbI_2_ beads (99.999% trace metals basis) were purchased from Sigma-Aldrich, which have good solubility in DMF. Around 60 μl of hot PbI_2_ precursor solution, which was pre-heated to 110 °C, was directly transferred by plastic (polypropylene) disposable pipettes from the bottle of heated PbI_2_ solution to the HTL-covered ITO substrates within 2 s. The spinning was quickly started at the speed of 6,000 r.p.m. after the injection of PbI_2_ solution. The as-fabricated PbI_2_ films were dried and annealed at 110 °C for 5 min. To fabricate 360-, 500- and 600-nm-thick MAPbI_3_ film, 600, 700 and 800 mg ml^−1^ PbI_2_ DMF precursor solutions were used with 70, 80 and 95 mg ml^−1^ methylammonium iodide (MAI) 2-propanol precursor solution, respectively. The MAI was synthesized from methylamine (40 wt% in H_2_O, Sigma-Aldrich) and hydroiodic acid (57 wt% in H_2_O, 99.95%, with stabilizer, Sigma-Aldrich) and the method was reported elsewhere^9^. The deposition of MAI layer was taken at 6,000 r.p.m. and from a hot precursor solution at 70 °C. The stacked precursor layers were annealed on the hotplate at 105 °C with a Petri dish covering them. The following deposition of phenyl-C61-butyric acid methyl ester, C_60_, 2,9-dimethyl-4,7-diphenyl-1,10-phenanthroline and Al layers were reported elsewhere^10^,^12^,^19^. The device working area was 7.25 mm^2^, defined by the overlap of ITO substrate and Al cathode.

### Film and device characterization

The microscope images of PbI_2_ layer spin coated at 70 and 110 °C were taken by an optical microscope Olympus BX61, with an integrated charge-coupled device (Photometrics, CoolSNAP-cf). X-ray diffraction pattern was obtained by a Rigaku D/Max-B X-ray diffractometer with Bragg–Brentano parafocusing geometry. A Co-Kα tube was equipped in the diffractometer with an emitting wavelength of 1.79 Å. The SEM images were taken from a Quanta 200 FEG environmental scanning electron microscope. The grain size was obtained by measuring the average diameter of the grains in the plane direction from SEM images. A Xenon lamp-based solar simulator (Oriel 67005, 150 W solar simulator) was used to produce the simulated AM 1.5G irradiation (100 mW cm^−2^), and the calibration of the light was carried out by a Si diode (Hamamatsu S1133) equipped with a Schott visible-colour glass filter (KG5 colour filter). The bias scanning rate was 0.13 V s^−1^ for the device *J–V* curve measurement. TAS measurement was performed by a LCR (inductance (L), capacitance (C), and resistance (R)) meter (Agilent E4980A) to obtain the devices' frequency-dependent capacitance and voltage-dependent capacitance, which was used for devices' tDOS derivation. The derivation procedure was reported elsewhere^4^. Impedance spectroscopy was also recorded by the LCR meter (Agilent E4980A) with home-made software. The devices were kept under 1 sun illumination at room temperature during the measurement. The recombination lifetime equates to the reciprocal of the angular frequency at the top of the arc in impedance spectra with the Nyquist plot^27^ (Supplementary Fig. 8), which has good agreement with the fitted results from recombination resistance and chemical capacitance (Supplementary Figs 9 and 10).

## Additional information

**How to cite this article:** Bi, C. *et al.* Non-wetting surface-driven high-aspect-ratio crystalline grain growth for efficient hybrid perovskite solar cells. *Nat. Commun.* 6:7747 doi: 10.1038/ncomms8747 (2015).

## Supplementary Material

Supplementary Information Supplementary Figures 1-10 and Supplementary References

## Figures and Tables

**Table 1:** Summary of the best device performance for the OTP devices with different HTLs and different thickness of active layers.

**HTL and active layer thickness**	***J***_**SC**_ **(mA cm**^−2^)	***V***_**OC**_ **(V)**	**FF** **(%)**	**PCE (%)**
PEDOT:PSS 400 nm MAPbI_3_	18.6	0.92	72.0	12.3
c-OTPD 360 nm MAPbI_3_	21.2	1.09	75.5	17.5
c-OTPD 500 nm MAPbI_3_	22.4	1.05	75.6	17.8
c-OTPD 600 nm MAPbI_3_	22.2	1.06	73.4	17.2
PTAA 500 nm MAPbI_3_	22.0	1.07	76.8	18.1
