# Supplementary material for: Non-wetting surface-driven high-aspect-ratio crystalline grain growth for efficient hybrid perovskite solar cells
Source: Nat Commun. 2015 Jul 20;6:7747. doi: 10.1038/ncomms8747 (PMC4518278; doi:10.1038/ncomms8747)
Supplement: Supplementary Information — Supplementary Figures 1-10 and Supplementary References [file ncomms8747-s1.pdf]

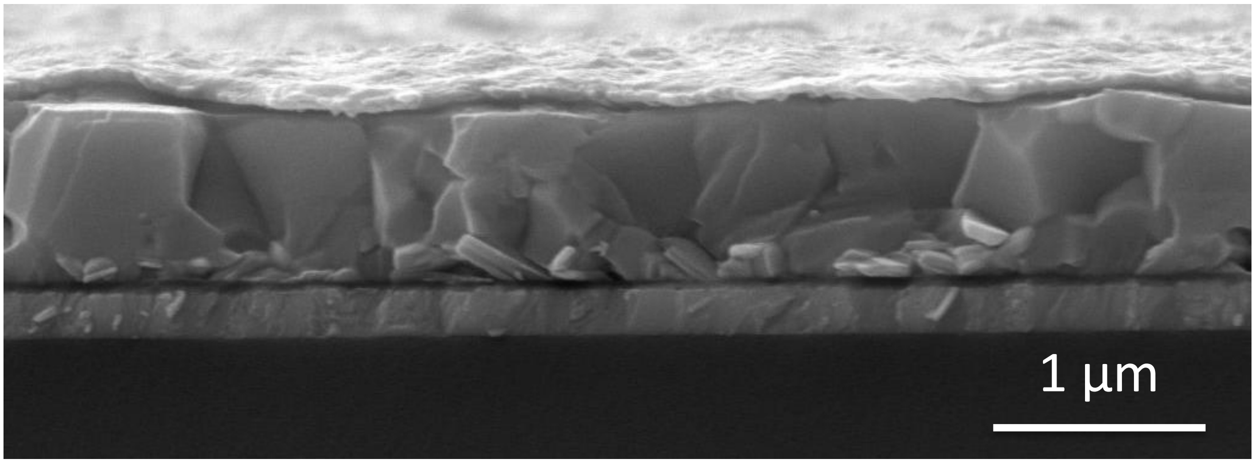

Supplementary Figure 1. Cross-section SEM image of solvent-annealed methylammonium lead triiodide ($MAPbI_3$) film on poly(3,4-ethylenedioxythiophene) polystyrene sulfonate (PEDOT:PSS). Supplementary Figure 1 shows the cross-section image of solvent-annealed $MAPbI_3$ film (1 µm thick) grown on PEDOT:PSS substrate. The method of solvent annealing used can be found elsewhere.[1] It is noted that although large grains comparable to the film thickness consist of film's most cross-section area, some small grains can be found in the film particularly close PEDOT:PSS side, which should result from the reduction of grain boundary (GB) mobility due to surface tension dragging force from wetting PEDOT:PSS substrate. In addition, the average grain size is still comparable to the film thickness.

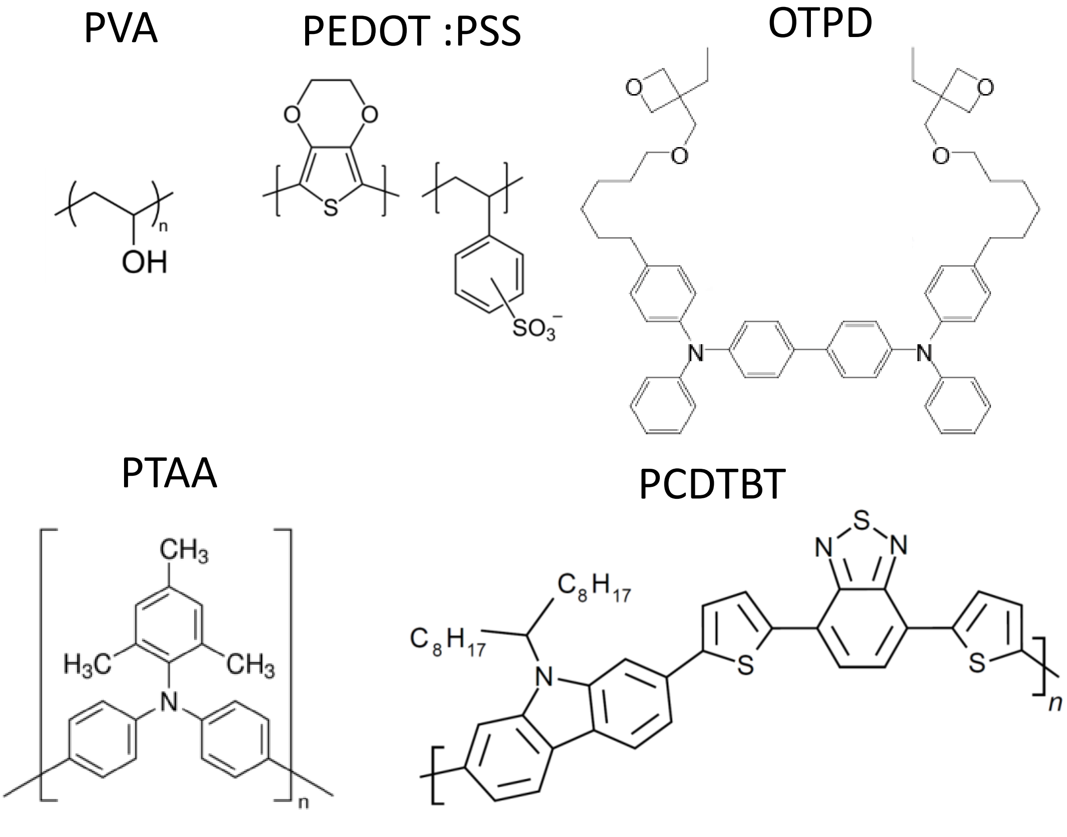

Supplementary Figure 2. Chemical structures of hole transport layer (HTL) materials: Polyvinyl alcohol (PVA), PEDOT:PSS, cross-linked N4,N4'-Bis(4-(6-((3-ethyloxetan-3-yl)methoxy)hexyl)phenyl)-N4,N4'-diphenylbiphenyl-4,4'-diamine (c-OTPD), Poly[bis(4-phenyl)(2,4,6-trimethylphenyl)amine] (PTAA), and Poly[N-9'-heptadecanyl-2,7-carbazole-alt-5,5-(4',7'-di-2-thienyl-2',1',3'-benzothiadiazole)] (PCDTBT) .

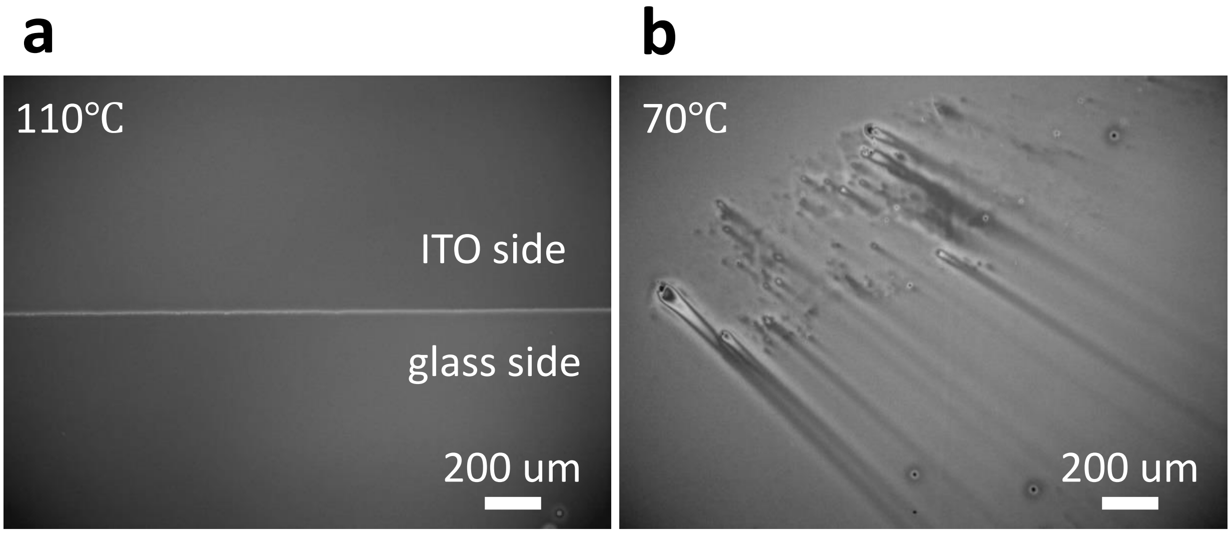

Supplementary Figure 3. Optical microscope pictures for PbI_2 film spun on c-OTPD for the PbI_2 solution preheated at (a) 110°C and (b) 70 °C, respectively. Supplementary Figure 3 indicates that in order to spin-coat a uniform PbI_2 layer on non-wetting substrate of c-OTPD, a high PbI_2 solution temperature (110°C) is needed for the quick drying of the solvent. A lower solution temperature (70 °C) usually yielded a non-uniform PbI_2 film with many spots.

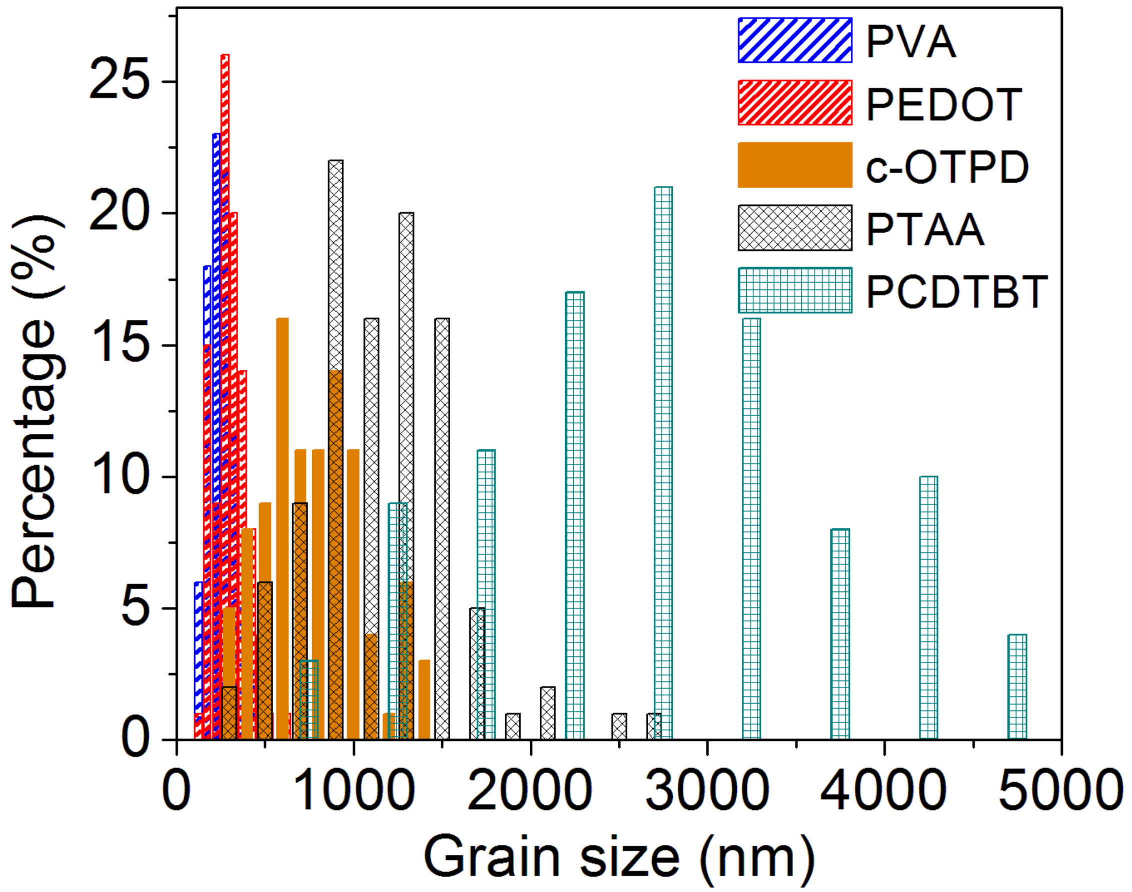

Supplementary Figure 4. Grain size distribution of the $MAPbI_3$ films grown on various HTLs. Supplementary Figure 4 shows the grain size distribution of the $MAPbI_3$ films grown on different HTLs. All the grain sizes have a Gaussian distribution. The average size for $MAPbI_3$ films grown on PVA, PEDOT:PSS, c-OTPD, PTAA and PCDTBT is 277 nm, 301 nm, 813 nm, 1154 nm and 2,751 nm, respectively.

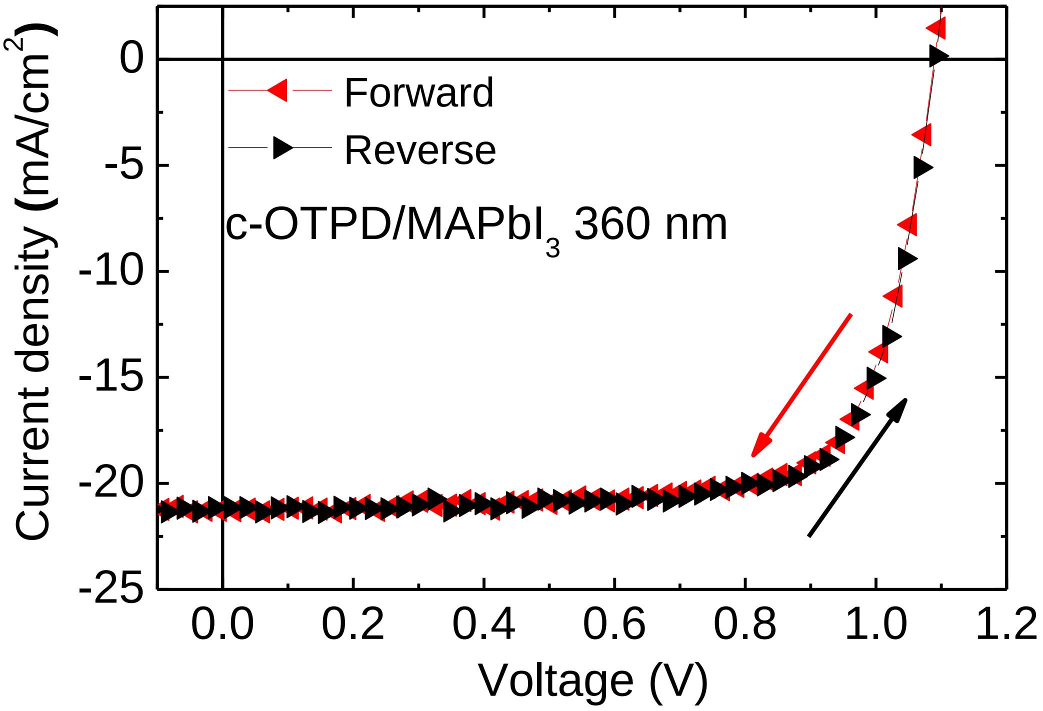

Supplementary Figure 5. J-V curves of the $MAPbI_3$ devices with different bias scanning direction. The HTL is c-OTPD. Supplementary Figure 5 shows the photocurrent hysteresis characterization by applying scanning bias from forward (1.2 V to -1 V) and reverse (-1 V to 1.2 V) direction at the rate of 0.13 V s^{-1}. The $MAPbI_3$ device has a c-OTPD HTL and 360 nm thick active layer. No obvious photocurrent hysteresis was observed by switching the scanning direction due to reduction of trap amount, which results from the large grain size on non-wetting c-OTPD and top passivation of phenyl-C_{61}-butyric acid methyl ester (PCBM). The reverse scanning yielded J_{SC} of 21.2 mA cm^{-2}, V_{OC} of 1.09, *FF* of 75.5% and PCE of 17.5%, while reverse scanning had J_{SC} of 21.3 mA cm^{-2}, V_{OC} of 1.10, *FF* of 73.8% and PCE of 17.2%.

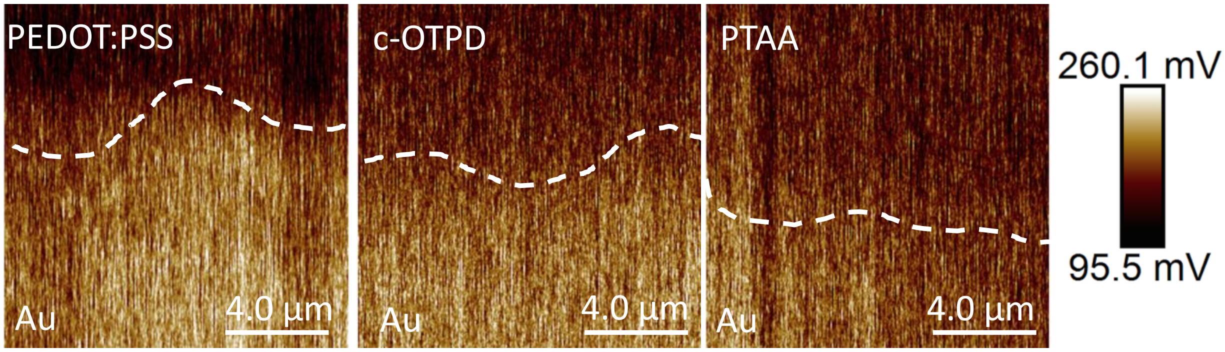

Supplementary Figure 6. Kelvin probe force microscopy (KPFM) images of three HTL layers by using thermal evaporated Au as a reference. Supplementary Figure 6 shows the surface potential of varied HTL polymer layers (PEDOT:PSS, c-OTPD and PTAA) by using Au as a reference. The PEDOT:PSS has the lowest work function (WF), while PTAA has the highest WF. The increase of device V_{OC} by switching HTL from PEDOT:PSS to c-OTPD and PTAA can be partially explained by the increase of HTL WF. The WF distribution in Figure 3h was extracted from Supplementary Figure 6.

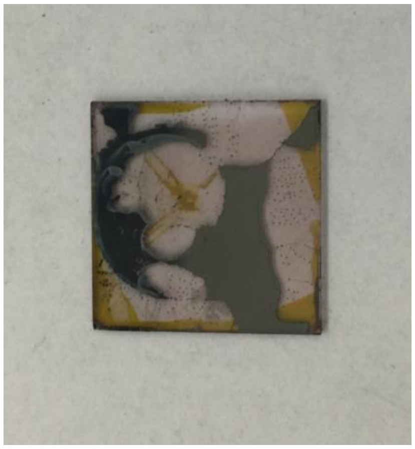

Supplementary Figure 7. Photograph of non-continuous $MAPbI_3$ film on PCDTBT substrate. Supplementary Figure 7 shows the typical $MAPbI_3$ film grown on PCDTBT substrate. The PCDTBT substrate is too hydrophobic, and thus it is difficult to reach 100% coverage of the $MAPbI_3$ film to the PCDTBT substrate, which makes PCDTBT substrate not suitable for device optimization.

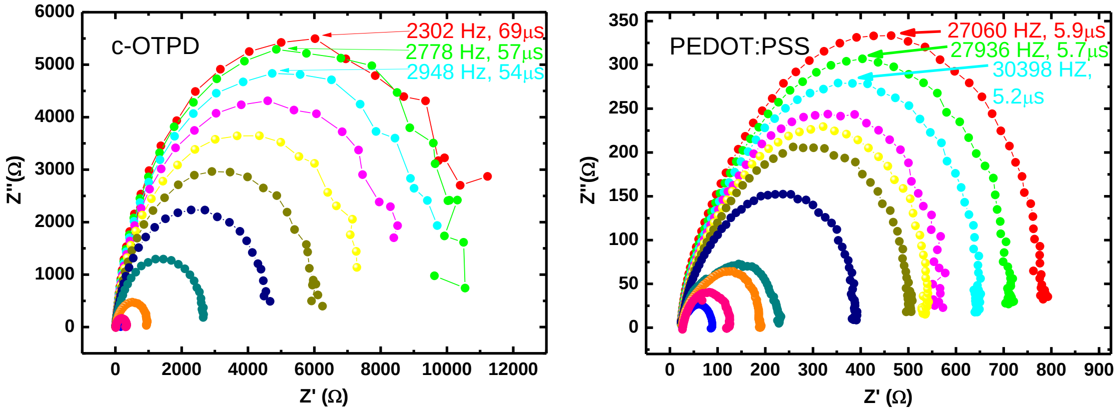

Supplementary Figure 8. Impedance spectra of the devices with c-OTPD and PEDOT:PSS HTLs.

Supplementary Figure 8 shows the impedance spectra with Nyquist plot for the device with c-OTPD and PEDOT:PSS HTLs. The recombination lifetime equates to the reciprocal of the angular frequency at the top of the arc in impedance spectra with the Nyquist plot [2].

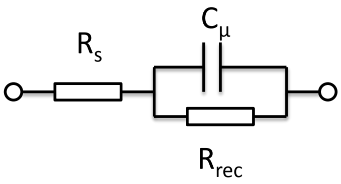

Supplementary Figure 9. R(CR) equivalent circuit for fitting the impedance spectra

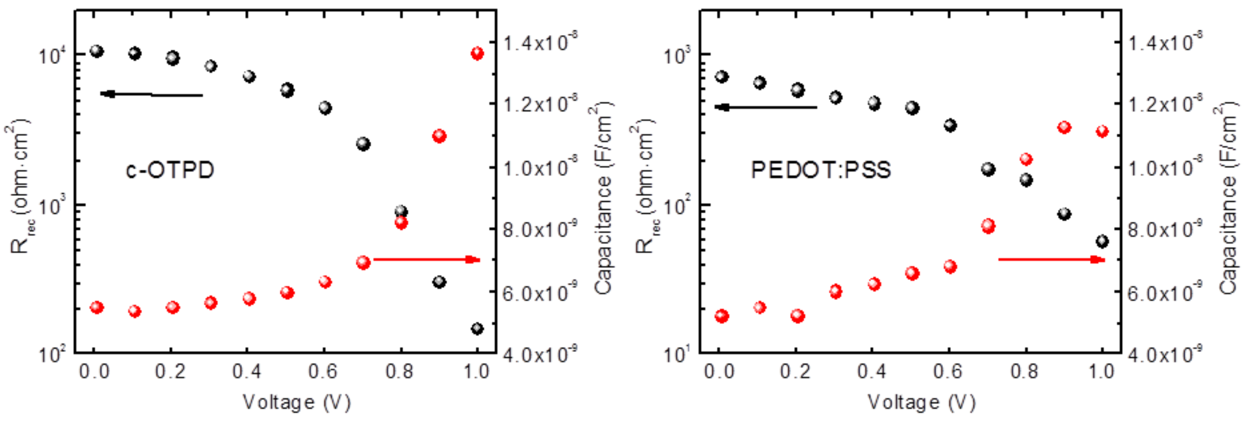

Supplementary Figure 10. The fitted recombination resistance (R_{rec}) and chemical capacitance (C_{μ}) for devices on c-OTPD and PEDOT:PSS substrate.

Supplementary References

1. Xiao, Z. *et al.* Solvent-Annealing of Perovskite Induced Crystal Growth for Photovoltaic Device Efficiency Enhancement. *Adv. Mater.* **26**, 6503-6509 (2014).

2 . Mora-Seró, I. *et al*, Impedance Spectroscopy Characterisation of Highly Efficient Silicon Solar Cells Under Different Light Illumination Intensities. *Energy Environ. Sci.*, **2**, 678–686 (2009).
